# Heterologous expression of *cry3Bb1* and* cry3* genes for enhanced resistance against insect pests in cotton

**DOI:** 10.1038/s41598-022-13295-x

**Published:** 2022-06-27

**Authors:** Muhammad Mubashar Zafar, Ghulam Mustafa, Fiza Shoukat, Atif Idrees, Arfan Ali, Faiza Sharif, Amir Shakeel, Huijuan Mo, Yuan Youlu, Qurban Ali, Abdul Razzaq, Maozhi Ren, Fuguang Li

**Affiliations:** 1 State Key Laboratory of Cotton Biology, Key Laboratory of Biological and Genetic Breeding of Cotton, The Ministry of Agriculture, Institute of Cotton Research, Chinese Academy of Agricultural Science, Anyang, 455000 Henan China; 2 Zhengzhou Research Base, State Key Laboratory of Cotton Biology, School of Agricultural Sciences of Zhengzhou University, Zhengzhou, 450000 China; 3 Center of Agricultural Biochemistry and Biotechnology, University of Agriculture, Faisalabad, Pakistan; 4 Guangdong Key Laboratory of Animal Conservation and Resource Utilization, Guangdong Public Laboratory of Wild Animal Conservation and Utilization, Institute of Zoology, Guangdong Academy of Sciences, Guangzhou, 510260 China; 5 Department of Plant Breeding and Genetics, University of Agriculture, Faisalabad, Pakistan; 6 FB Genetics, Four Brothers Group, Lahore, Pakistan; 7 Institute of Molecular Biology and Biotechnology, The University of Lahore, Lahore, Pakistan; 8 University Institute of Physical Therapy, The University of Lahore, Lahore, Pakistan; 9 Department of Plant Breeding and Genetics, Faculty of Agriculture, University of the Punjab, Lahore, Pakistan

**Keywords:** Biological techniques, Biotechnology, Molecular biology

## Abstract

Transgenic technology played a crucial role in developing insect-resistant plants resulting in the reduced application of pesticides. This article reports the expression of two cry proteins (*Cry3Bb1 and Cry3*) in cotton for enhanced resistance against chewing insect pests. The aforementioned genes were synthetically developed and were cloned under appropriate regulatory sequences followed by transformation into Eagle-2 genotype (*Gossypium hirsutum*) of cotton through shoot apex-cut Agro-infiltration. The transgene integration was validated by polymerase chain reaction using primers flanking the aforementioned cry genes. Transgene expression was assessed by qRT-PCR using *GADPH* as a reference gene. The relative fold expression analyses revealed the highest expression of the transgene(s) in M1 plants, which is a 4.5-fold expression (*Cry3* + *Cry3Bb1*) followed by M3 (fold expression, 3.0) (*Cry3Bb1*) and M2 (fold expression, 2.5) (*Cry3*) transformants of cotton. The confirmed transgenic plants were exposed to insect pests, pink bollworm (*Pectinophora gossypiella*), and army bollworm (*Helicoverpa armigera*). Bioassay results revealed that 60% mortality was observed against pink bollworm, and 75% mortality was observed against army bollworm in transgenic plants containing both *Cry3Bb1* and *Cry3* genes (M1 transgenic plants). In M2 transgenic plants containing only the *Cry3Bb1* gene, the mortality was observed to be 40% in the pink bollworm population, whereas 45% mortality was observed in the army bollworm population. In the case of M3 transgenic plants containing single gene-*Cry3*, the mortality was 20% in the pink bollworm population, whereas 30% mortality was observed in the army bollworm population. Almost no mortality was observed in non-transgenic Eagle-2 control plants. Hence, the developed cotton transformants have improved resistance against chewing insect pests.

## Introduction

Cotton (*G. hirsutum*) is a major agro-industrial crop grown worldwide for oil and fiber purposes^1^. The production of cotton is negatively affected by both biotic and abiotic stresses^1^. Different studies suggested that insect pests and diseases bring about 15–30% yield losses, and sometimes these losses can reach up to 50% due to direct injury and plant disease transmission^2^. The cotton fields are attacked by insect pests such as pink bollworm and army bollworm. The intensive application of chemical pesticides results in pesticide resistance in cotton pests, as well as these are also destructive to the environment and human health^1^. Pesticide applications also have negative impacts on beneficial insects^3^. A soil-borne bacterium, *Bacillus thuringiensis* (Bt), produces various insecticidal proteins (cry toxins), which have been used widely against many pests and until now, numerous cry toxins have been transformed in cotton^4^. Bt cotton reduces the pest burden without harming human health and the environment. Globally, the (Bt) cry3 proteins are toxic to several agricultural pests of the order Coleoptera, Lepidoptera, and Diptera^5^.

Despite the proven substantial protective effects of transgenic Bt-cotton plants against insect attack, this technology still needs improvement^6,7^**.** For instance, gene pyramiding involves defense strategies against insect pests^8^. The overproduction or multi-gene expression involved in insect defense can be a good alternative to reduce the pest attack and the development of insect resistance to cry toxins^8^.

It is interesting to mention that *cry3Bb1* and *cry3* have been successfully reported in several other crops, such as maize^9^, poplar^10^, eggplant^11^, and tobacco^12^, but it is hardly seen that these genes have been transformed into cotton. These genes have been proven effective strategy against insect pests in the aforementioned crops.

The early Bt genes transformed into maize were *Cry1Ab*, *Cry1Ac* and *Cry2A* ^13^. Subsequently, *Cry34/35Ab1* and *Cry3Bb1* genes were transformed for the management of closely related pest species^14^. Earlier, the stacking of multiple genes is reported in various crops, for example, a maize variety expressing five and six Bt genes (*Cry1Ab*, *eCry3.1Ab*, *Cry1Fa2,* *mCry3A*, and *Vip3Aa20*) and (*Cry2Ab2*, *Cry1F*, *Cry1A.105*, Cry*35Ab1, Cry34Ab1* and *Cry3Bb1*) respectively^15^. *Cry3Bb1* is one of the most commonly used Bt toxins in GM maize, has good insecticidal activity against Colorado potato beetle (*Leptinotarsa decemlineata*) and even better activity against western corn rootworm (*D. v. virgifera*)^16^. In eastern North Dakota (United States), the total feeding injury and population level of western corn rootworm were the lowest on *Cry3Bb1* + *Cry34/35Ab1* hybrids than on Bt maize producing either *Cry3Bb1 or Cry34/35Ab1* protein alone^17^. In another study, the *Cry3Bb1* gene expressed in maize showed resistance against *Ostrinia furnacalis*^18^.

In this proposed study, *cry3Bb1* and *cry3* have been tried in cotton against pink bollworm and army bollworm and produced a substantial effect. This study provides a new insight into how these *cry* genes can contribute significantly against insect pests in cotton.

## Results

### Development of plant transformation vector harboring double Cry gene(s)

**Figure 1:**
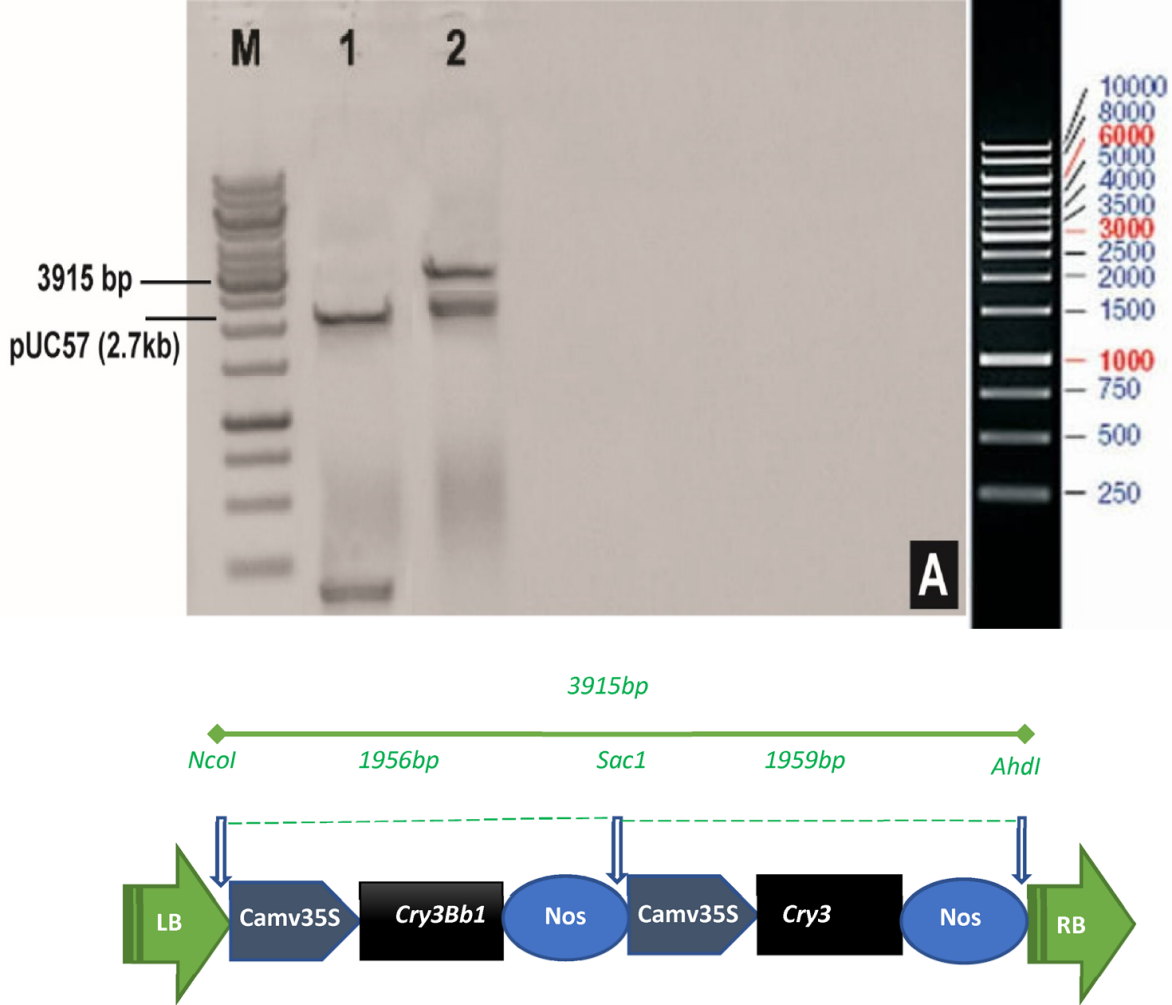
Restriction analysis of puc57 vectors containing the *cry3Bb1* and *cry3* gene cassette (total 3915 bp size) (**A**). *NcoI* and *AhdI* restriction analysis of the *cry3Bb1* and *cry3* gene cassette. M: 10 kb ladder. Lane 1: control of pUC57. Lane 2: *cry3Bb1* and *cry3* gene cassette. (**B**) Schematic representation of both gene cassette containing *cry3Bb1* and *cry3* genes.

Two synthetic genes (*Cry3Bb1* and *Cry3*) were synthesized from Synbio after codon optimization. Standard cloning was received in pUC57 cloning vector, wherein they were confirmed with *AhdI, SacI* and *NcoI* restriction endonucleases (Fig. 1a). The expected size of the cassette 3915 bp containing *Cry* genes was observed in Fig. 1a. A schematic diagram of the gene cassette carrying *Cry3Bb1* and *Cry3* genes is presented in Fig. 1b.

**Figure 2:**
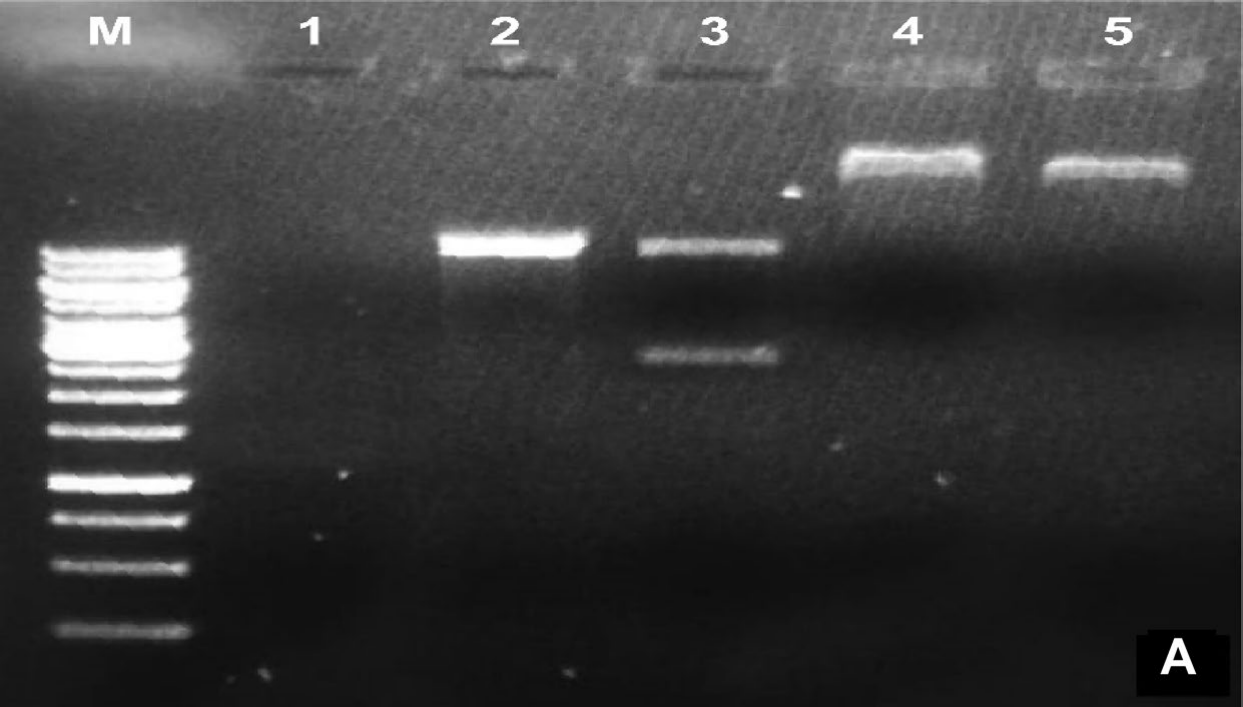

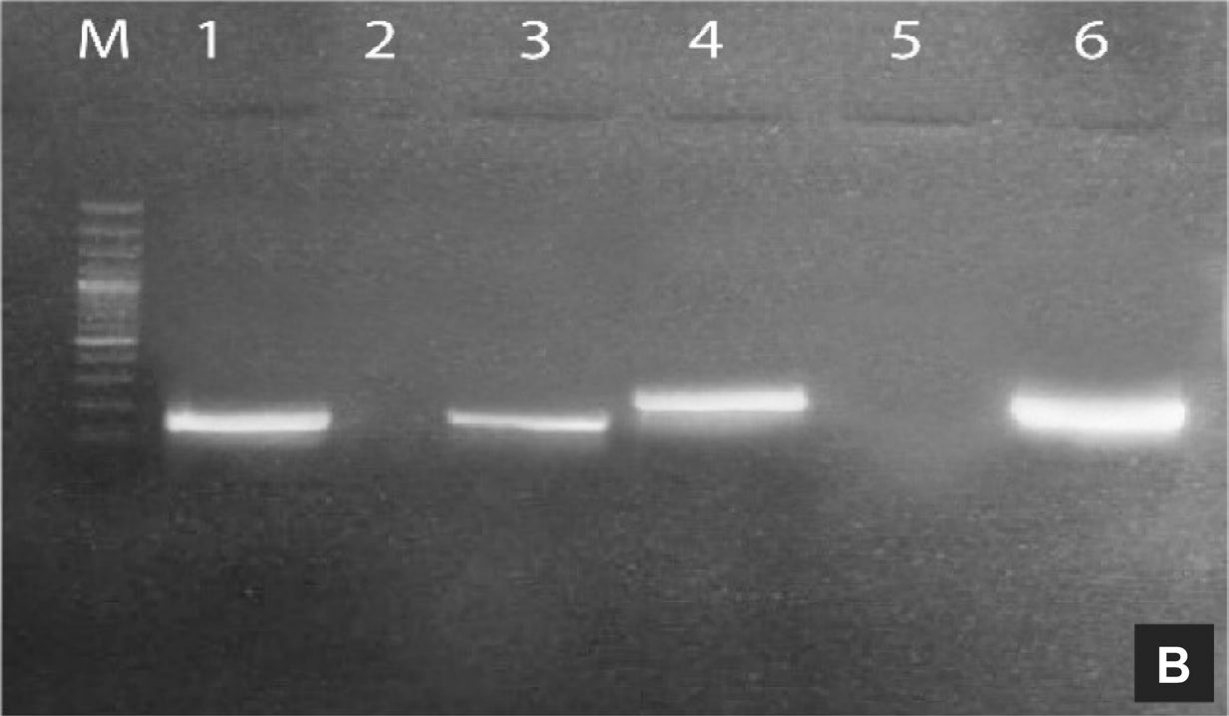
Restriction digestion of both *cry* gene. (**A**) *NcoI* and *AhdI* restriction of pCAMBIA1301 vector containing *cry* genes cassette; M: 10 kb molecular weight marker, Lanes 1: shows blank, Lane 2: shows pCAMBIA1301, Lane 3: shows ligation without ligase, Lane 4, 5: shows ligation of cry genes with pCAMBIA1301. (**B**) PCR amplification of both *cry* genes. M: 10 kb molecular weight marker, Lanes 1, 2: show *cry3Bb1* gene (459 bp), Lane 3, 5: show *cry3* gene (562 bp), Lane 4: shows a blank.

The developed Cry gene(s) cassette was cloned in plant expression vector pCAMBIA1301 and was confirmed by restriction analysis with *NcoI* and *AhdI* (Fig. 2a). Cloning of the crystal protein genes (*Cry3Bb1* and *Cry3*) was also confirmed by PCR (Fig. 2b).

### Genetic transformation of cotton through *Agrobacterium tumefaciens* harboring double Cry gene(s)

**Figure 3:**
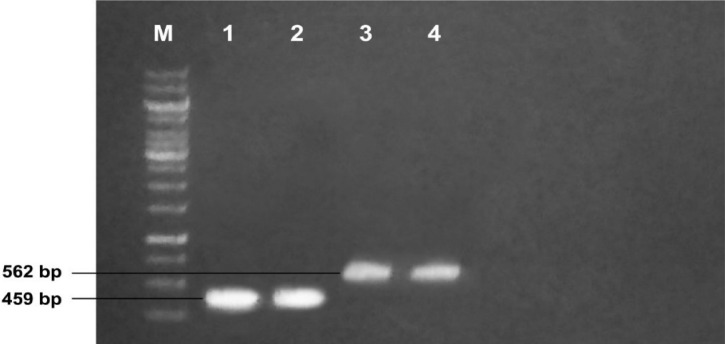
PCR amplification of both *cry* genes from transformed *A. tumefaciens* with the corresponding pCAMBIA1301-*cryCassette* constructions. M: 10 kb molecular weight marker, Lanes 1, 2: show *cry3Bb1* gene, Lane 3, 4: show *cry3* gene.

The discrete colonies of *A. tumefaciens* strain LBA4404 transformed with crystal protein genes were randomly selected by colony PCR using gene-specific primers for *cry3Bb1* and *Cry3* genes. The PCR product was resolved on 1.5% agarose gel. The PCR product exhibited the expected size of the amplicon, 459 bp and 562 bp for *Cry3Bb1* and *Cry3* genes, respectively (Fig. 3).

**Figure 4:**
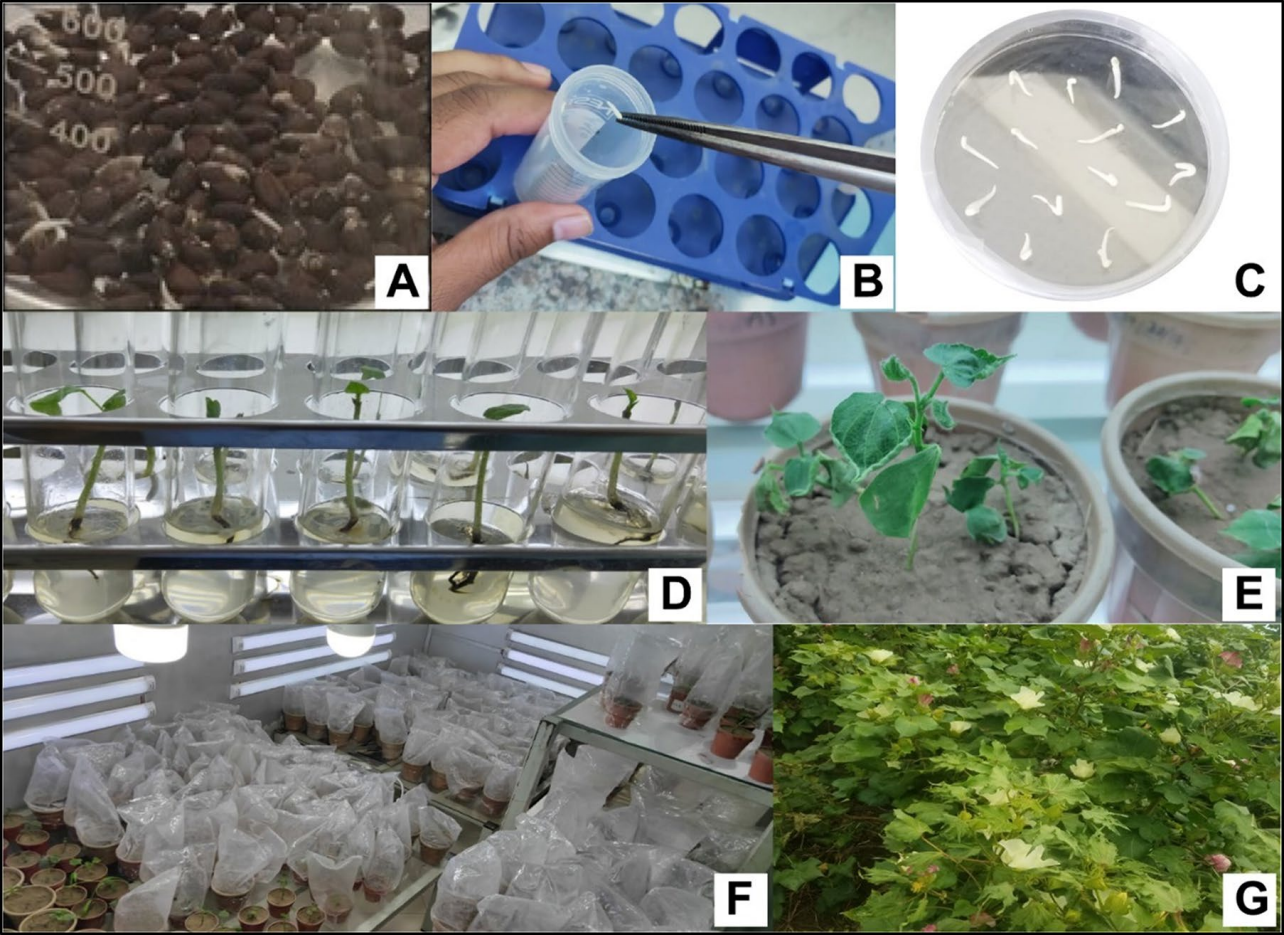
A schematic procedure of gene transformation in cotton; (**A**) soaking of seeds, (**B**) co-cultivation of embryos into MS media, (**C**) shifting of embryos on MS plates, (**D**) shifting of embryos into MS tubes, (**E,F**) shifting of plants into the pots, (**G**) shifting of plants into the field.

The seeds of *G. hirsutum* variety Eagle-2 were delinted, surface sterilized using 5% HgCl_2_ and 1–2 drops of 2.5% SDS and incubated at 30 °C for 48 h. The germinated seedlings were used for transformation using the shoot apex cut method. The seedlings were incised and inoculated with *A. tumefaciens* cells containing both *Cry3Bb1* and *Cry3* genes (Table 1). The germination index and transformation efficiency of both control and transgenic plants were found to be 77.14% and 1.56%, respectively (Tables 2 and 3). A schematic transformation procedure is given in Fig. 4.

**Table 1:** Numerical data for transformation experiments.

Exp. No	No. of embryos isolated	Agrobacterium treated embryos	Embryos on MS plates	Died	Selection tubes	Plantlets died	Plants transferred to pots	plants died in pots	Plants shifted to greenhouse
1	110	104	104	96	8	4	4	1	3
2	115	108	108	103	5	1	4	2	2
3	120	109	109	101	8	3	5	2	3
4	101	94	94	91	3	1	2	0	2
5	98	96	96	93	3	1	2	1	1
6	160	155	155	150	5	3	2	0	2
7	190	179	179	173	6	1	5	1	4
Total	894	845	845	807	38	14	24	7	17

**Table 2:** Germination Index.

No. of petri plates	Total seeds	No. of germinated seeds	No. of ungerminated seeds	Germination index
1	35	27	8	77.14%
2	35	30

**Table 3:** Transformation efficiency.

Agrobacterium treated embryos	Control plants	Plants shifted to green house		Transformation efficiency	
		Control plants	Experimental	Control plants	Experimental
894	50	24	15	48%	1.56%

### Molecular analyses of putative transformants of cotton

**Figure 5:**
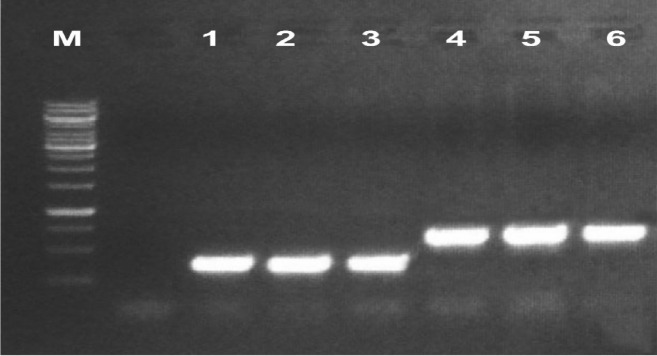
PCR amplification of both *cry* genes from transformed cotton. M: 10 kb molecular weight marker, Lane 1, 2, 3: show *cry3Bb1* gene. Lanes 4, 5, 6: show *cry3* gene.

Fresh leaves were taken from the putative transformants of cotton. Total cellular DNA was isolated and was subjected to polymerase chain reaction using primers flanking the aforementioned cry genes. The PCR amplification resulted in product sizes of 459 bp and 562 bp for Cry (*Cry3Bb1* & *Cry3*) genes, respectively (Fig. 5).

**Figure 6:**
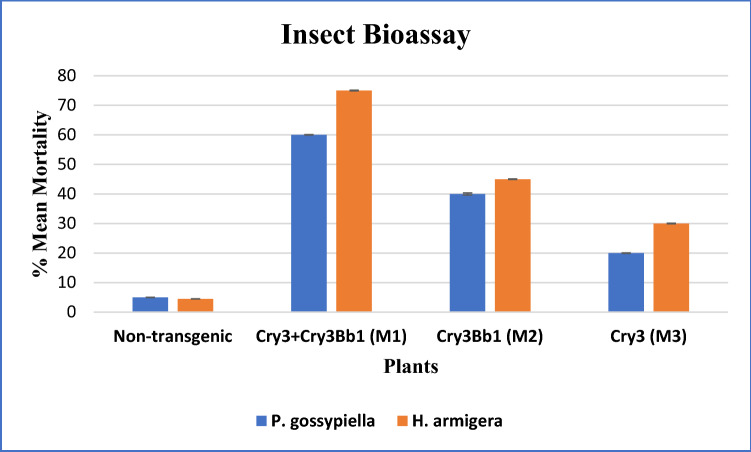
Relative expression of *cry* genes in three transgenic cotton plants (M1, M2, and M3). The relative expression of *cry* genes, analyzed in different plants shown in the figure was calculate according to the 2^(−ΔΔCt)^ method using GAPDH as internal control reference gene for normalization.

Total cellular mRNA was isolated from the leaves of putative cotton transformants and was used to synthesize cDNA. The resultant cDNA was used in order to track the expression of the transgenes (*Cry3Bb1* and *Cry3*) using SYBR Green Supermix (Thermo Scientific, Cat#K0221) in the qRT-PCR assay. Relative expression analyses were carried out for the various transformants, whereas the *GAPDH* gene was used as an internal control for the normalization of reaction. Expression of both Cry genes was observed to be higher in M1 transgenic plants as compared to M2 and M3 transgenic plants, whereas no expression was detected in the non-transgenic cotton plants (Fig. 6).

### Evaluation of transgenic cotton plants for resistance against insects.

**Figure 7:**
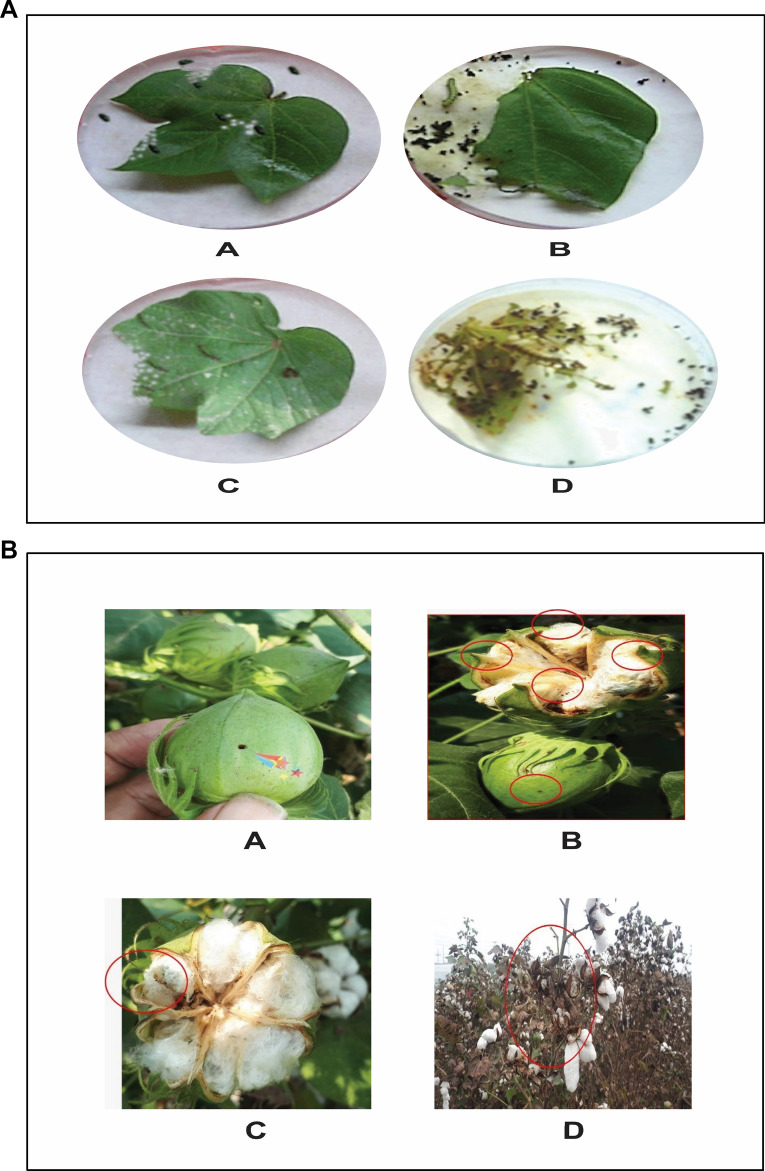
(**A**) Cotton leaf mortality assay of army bollworm in different transgenic and non-transgenic cotton plants. Non-transgenic cotton plant leaf almost completely infected by army bollworm. (**A**) Transgenic cotton plant leaves containing both Bt-gene (*cry3Bb1* and *cry3*), (**B**) shows transgenic plants containing *cry3Bb1*, (**C**) shows transgenic plants containing *cry3*, and (**D**) shows non-transgenic plants. (**B**) Pink bollworm mortality assay in transgenic and non-transgenic cotton plants. Non-transgenic cotton plant leaf almost completely infected by pink bollworm. (**A**) Transgenic cotton plant leaves containing both Bt-gene (*cry3Bb1* and *cry3*), (**B**) shows transgenic plants containing *cry3Bb1*, (**C**) shows transgenic plants containing *cry3*, and (**D**) shows non-transgenic plants.

The whole transgenic and non-transgenic plants were selected from the field for bioassay analysis. Transgenic plants (M1, M2 and M3), engineered with *Cry3Bb1* and *Cry3* genes, as well as non-transgenic plants, were exposed to pink bollworm and army bollworm. 5–7 3rd instar larvae were used in triplicates, and infestation data were recorded. On the third day of infection, 60% mortality was observed against pink bollworm, and 75% mortality was observed against army bollworm in transgenic plants containing both *Cry3Bb1* and *Cry3* (M1 transgenic plants). In M2 transgenic plants containing only *Cry3Bb1*, the mortality was observed to be 40% against pink bollworm, whereas 45% mortality was observed against army bollworm. In the case of M3 transgenic plants containing single gene-*Cry3*, the mortality was observed to be 20% against pink bollworm, whereas 30% mortality was observed against army bollworm. Almost no mortality was observed in non-transgenic Eagle-2 control plants (Fig. 7a,b).

**Figure 8:**
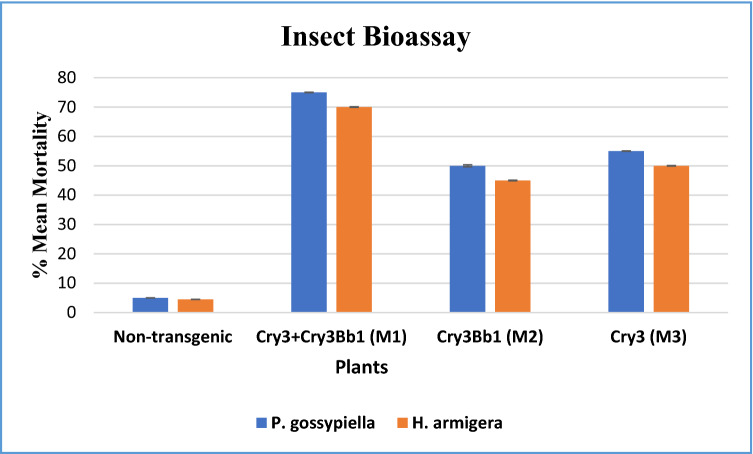
Mortality analysis against pink bollworm and army bollworm*.* Two-way ANOVA showed the significance of the data compared to the non-transformed plants.

Two-way ANOVA showed the significance of our data at *P* ≤ 0.0001 (Fig. 8).

## Discussion

Cotton being the leading fiber as well as an oil crop has great economic importance, and due to increased population pressure, its demand is also increased. So continuous improvement in the cotton crop against both biotic and abiotic stresses is important for cotton-growing countries. The invasive cotton pests like pink bollworm and army bollworm limit cotton production badly, so the transformation of novel genes is necessary for the development of resistance against these pests. For this purpose, a novel strategy was used; therein, a gene cassette was developed. The cassette contains two *cry* genes from *B. thuringiensis*. In the non-transgenic cotton variety Eagle-2, this cassette was transformed for the development of resistance against pests as reported in maize and tobacco. As reported earlier, the initial screening of putative cotton plants was performed with hygromycin^19^. These transgenic plants were confirmed by PCR as reported^20^. The qPCR was performed for PCR verified transgenic plants to determine mRNA expression^21^. Ultimately, to ascertain the effectiveness of transgene, an insect bioassay was performed against different target coleopteran and lepidopteran insects^22^.

In plants, several approaches are used for the insertion of a foreign gene, but *Agrobacterium*-mediated transformation is widely adopted in plant biotechnology. For instance, cotton shoot apex *Agrobacterium*-mediated transformation is more acceptable to exploit the transgenic technology in various cultivars^23,24^. From the early twentieth century, *Agrobacterium* and related species were known as plant pathogens. But with the continuous extensive research on *Agrobacterium,* it has been found that this bacterium can be used as a “natural genetic engineer”. Later studies showed that the involvement of host range, alterations in plant culture media and regeneration conditions are equally important along with improvement in a bacterium or host–pathogen^25^. The efficiency of *Agrobacterium*-mediated transformation depends on the type of promoter, *Agrobacterium* suspension medium, co-cultivation time and seed germination index^26^. Our study revealed a 77.14% seed germination index as well as 48% controlled and 1.56% experimental transformation efficiency.

Western corn rootworm is a serious threat to the maize crop in the USA. The upsurge in field evolved resistance against single Bt toxins reduced the efficacy of single gene transformation technology. Therefore, double or gene stacking technology is adopted to minimize the risks of resistance among various devastating pests. The transformation of the *Cry3Bb1* gene is effective against western corn rootworms^27^. The transformation of both *Cry3Bb* and *Cry3* in maize has produced many folds of higher expression against rootworms and leaf-feeding beetles^5^.

In this experiment, real-time qPCR assays were carried out to examine the mRNA expression of *cry* genes in transgenic plants. Compared to controls, the M1 plants revealed higher relative fold expression of 4.5 against pink bollworm and 4.3 against army bollworm than M2 (2.5 against pink bollworm and 2.2 army bollworm) and M3 (3.0 against pink bollworm and 2.5 against army bollworm).

Insect bioassays were conducted on the whole plant in the field. The data regarding infestation were recorded, and mortality of larvae was noted after three days in transgenic plants having both Bt Cry-genes, whilst no mortality was found in negative control cotton plants (Fig. 8). The larvae of pink bollworm and army bollworm were released on freshly growing leaves of the cotton plants. Transgenic cotton plants M1 exhibited 60% resistance towards pink bollworm whereas 75% resistance towards army bollworm for 3 days. In contrast, M2 transgenic plants revealed 40% and 45% resistance to pink bollworm and army bollworm. The transgenic plants M3 showed 20% and 30% resistance towards pink bollworm and army bollworm for 3 days. Our results suggested that stacking Bt Cry-genes is an efficient approach for managing various insect pests and minimizing the risk of resistance evolution in insects against transgenic cotton cultivars.

## Materials and methods

### Plant materials

The genotype Eagle-2 (*Gossypium hirsutum* L.) was used for the expression of two *cry* genes (*cryBb1 and cry3*). The healthy seeds of Eagle-2 were obtained from Four Brothers Seeds Multan-Pakistan and were grown at the research farm of Four Brothers Lahore-Pakistan.

### Development of synthetic cry genes

Gene sequences of selected *cry* genes (*cry3*, accession no. AY572010.1; *cry3Bb1*, accession no. spIQ06117I1-652) were retrieved from NCBI and, after codon optimization, were synthesized by [BIOBASIC, CANADA]. The synthetic double *cry3Bb1* gene and *cry3* gene cassette (total 3915 bp) were cloned into the *NcoI, SacI* and *Ahd1*(restriction endonuleases) restriction sites of pUC57 vector. All genes were under regulation of *CaMV35S* promoter, and *Nos* terminator was added at the end of these genes (Fig. 1).

### Development of plant transformation vector

The pUC57 vector carrying *cry3Bb1* and *cry3* genes cassette was transformed into the top 10 *E. coli* competent cells by using the heat shock method and selected on LB media containing ampicillin (50 µg/mL) and tetracycline (50 µg/mL). The Gene Jet plasmid DNA isolation kit (Thermo Scientific Vilnius, Lithuania, Cat#K0503) was used following the manufacturer’s instructions. For the confirmation of *cry3Bb1*and *cry3* genes, restriction digestion was done by using *NcoI* and *AhdI* enzymes. A 3.9 Kb DNA fragment of cry*3Bb1,* and *cry3* cassette were observed on 0.8% agarose gel. For the purification of eluted fragments, Gene JET Gel Extraction Kit (Thermo Scientific Vilnius, Lithuania, Cat#K0503) was used. The purified DNA fragment (cry*3Bb1* and *cry3* genes) was ligated into pCAMBIA1301 (plant expression vector), pre-digested with the corresponding restriction enzymes. A 10 Kb DNA marker (250 bp to 10 Kb) (GeneRuler, Thermo Scientific, Cat#SM0311) was used to assess the size of the resolved DNA fragments.

To confirm the ligation of the aforementioned cry genes in pCAMBIA1301 vector, restriction digestion was done with *NcoI* and *AhdI* enzymes. Further, cloning of these genes was also confirmed by PCR using gene(s) specific primers given in Table 4.

**Table 4:** Sequences of the primers used for the confirmation of cloning of the desired genes in final transformation vector pCAMBIA1301.

Primers	Sequence
GADPH-F	TGATGCCAAG GCTGGAATTGCTT
GADPH-R	GTGTCGGATCAAGTCGATAACACGG
Cry3Bb1-F	CCTTCTTTCGCCGTTTCCAA
Cry3Bb1-R	GGAAGGCCTAGTCTCAACGT
Cry3-F	CGTTCAATACCCTCTTGCCG
Cry3-R	AAATGGGATTCAGCCTGGGA
VirG-F	GAATACCTTACGATCCACGCC
VirG-R	GCGAAACCTGCACGTCCGCG
Cry3Bb1qRT-F	AGGACCATTGCTAACACCGA
Cry3Bb1qRT-R	GGAGGAAGCTGATCGATGGA
Cry3qRT-F	TACGGAGAGGAGTGGGGTTA
Cry3qRT-R	AGACCGACGTTGTACCACTT

Following conditions were used for the amplification of cry genes; Initial denaturation (94 °C for 3 min), denaturation (94 °C for 45 s), annealing (59 °C for 50 s), extension (72 °C for 1:30 min) and final extension (72 °C for 10 min). By electroporation, the confirmed plasmids were transformed into *A. tumefaciens* strain LBA4404 competent cells^28^. The YEP media (Peptone 10 g/L, Yeast extract 10 g/L, Sodium chloride 5 g/L, pH 7.5) containing Kanamycin (50 mg/ml) and Rifampicin (50 mg/ml) were used for the selection of recombinant *Agrobacterium* cells. Colony PCR was performed for the selection of desired *Agrobacterium* clones.

### Genetic transformation of selected cotton genotype

The surface sterilized seeds of Eagle-2 (upland cotton) were incubated in the dark at 30 °C for 48 h. The shoot apex cut method was used for the co-transformation of germinated seedlings. Before incubation, the isolated embryos were injured at the shoot apex and were treated with *Agrobacterium* strain LBA4404 harboring the pCAMBIA1301-cry. The cultures were incubated for 1 h. at 28 °C by placing explants on MS solid medium supplemented with (MS 4.4 g/L, Sucrose 30 g/L, Phytagel 2.4 g/L). After 48–72 h. of incubation, the embryos were cultured on MS media supplemented with cefotaxime (500 mg/ml), followed by screening in MS tubes containing hygromycin (25 mg/ml) for 1.5 months. After the screening, cotton plants were transferred into pots having an equal proportion of sand, clay and peat moss (1:1:1). Subsequently, the putative transgenic plants were shifted to greenhouses at Four Brothers Genetics Inc. for acclimatization and hardening. Thereafter molecular analyses were performed in order to assess for transgene integration and expression. The numerical data of genetic transformation was recorded in Table 1.

### Detection of the double cry gene integration into cotton genome

Leaves (usually third leaf) were harvested from putative transgenic plants for the isolation of DNA for PCR-based screening of *Cry3Bb1* and *Cry3* gene(s) integration. For this purpose, the PCR master mix kit (Thermo Scientific, cat#K1081) was used with specific primers. To nullify the *Agrobacterium* contamination, the amplification of *virG* gene was also performed using a specific set of primers from the *vir* region.

### Expression analysis of putative transformants of cotton

The Agilent kit (Agilent Technologies, Santa Clara, USA, Cat #5185-6000) was used for RNA isolation from putative cotton transformants. The Nano Drop ND-1000 spectrophotometer (at 260 and 280 nm) was used for the quantification of RNA. By using the first strand cDNA synthesis kit (Thermo Scientific, Cat #K1632), the cDNA was prepared from the DNase-treated total RNA. This cDNA was stored at −20 °C.

The qPCR was performed for expression analysis of transgenes with specific primers in triplicates with a Product size of < 200 bp using Maxima SYBR Green/ROX (Thermo Scientific, Cat#K0221). The reaction mixture of 20 µl with 1 µl of 10 pmol reverse and forward primers, 5 µl of Maxima SYBR Green/ROX qPCR Master Mix (2×) and 1 µl (50 ng/µl) of cDNA. The primer sequences for the amplification of both genes are given in Table 4. Relative expression was determined according to the 2 ^(−ΔΔCt)^ method using GAPDH primers as an internal control (reference gene) for normalization in reaction. All of the assays were carried out in triplicates.

### Insect bioassays

Insect bioassay was executed to assess the toxic impacts of transgenic and non-transgenic cotton plants against larvae of pink bollworm and army bollworm. The expanded fresh leaves of PCR positive plants were identified and placed in a petri dish for bioassay. 5–7 larvae of army bollworm of 3rd instar were used per leaf in triplicate. Similarly, pink bollworms of the 3rd instar were released on the transgenic and non-transgenic plants in the field. The effectiveness of transgenic plants against pink bollworm and army bollworm was assessed. The two-way ANOVA was used for statistical analysis. For 3 days, the mortality rate was continuously recorded using the following equation;

$$ \% {\text{ Mortality }} = {\text{ No of dead larvae}}/{\text{Total no}}.{\text{ of larvae}} \times {1}00. $$

### Research involvement with human participants/ or animals

The research does not involve human and animal participants.

### Informed consent

I have read and I understand the provided information and have granted permission to ask the questions. I understand my participation in the research and liable to produce the given information at any stage. The leaves of the plants were taken with the permission of FB Genetics Four Brothers Group Lahore.

## Conclusion

The simultaneous expression of both *Cry* genes was assessed against various insect pests. The transgenic plants containing two Bt *Cry-*genes were compared with non-transgenic plants. The mortality of pink bollworm and army bollworm was observed considerable in transgenic plants harboring both *Cry3Bb1* and *Cry3* (M1) genes as compared to M2, M3 and negative control. The insertion of these Bt genes in cotton will attract the attention of the farmers who are walking out from the major cotton-producing countries around the world. The transformed genes used as a combining strategy, which are hardly seen in transgenic cotton plants, will help reduce the insect attack leading to the improvement in cotton yield.

## Data Availability

The datasets generated and/or analysed during the current study are available in the “[NCBI] repository (https://www.ncbi.nlm.nih.gov/search/all/?term=+AY572010.1)”.
